# Thermal Mechanical Bending Response of Symmetrical Functionally Graded Material Plates

**DOI:** 10.3390/ma16134683

**Published:** 2023-06-28

**Authors:** Mengna Han, Zichan Li, Zhicheng Huang, Xingguo Wang, Wenjie Gao

**Affiliations:** College of Mechanical and Electronic Engineering, Jingdezhen Ceramic University, Jingdezhen 333001, China; m15214787876@163.com (M.H.); 13017234103@163.com (Z.L.); wangxingguo@jci.edu.cn (X.W.); gaowinjie@jci.edu.cn (W.G.)

**Keywords:** functionally graded materials, symmetrical sandwich plate, thermo-mechanical bending, deflection and stress

## Abstract

This paper investigates the thermal mechanical bending response of symmetric functionally graded material (FGM) plates. This article proposes a thermodynamic analysis model of both the FGM plate and FGM sandwich plate, and the model only involves four control equations and four unknown variables. The control equation is based on the refined shear deformation theory and the principle of minimum potential energy. The Navier method is used to solve the control equation. According to the method, numerical examples are provided for the thermo-mechanical bending of the symmetric FGM plate and FGM sandwich plate under a simply supported boundary condition, and the accuracy of the model is verified. Finally, parameter analysis is conducted to investigate the effects of the volume fraction index, side-to-thickness ratio, thermal load, and changes in core thickness on the thermal mechanical bending behavior of the symmetric FGM plate and FGM sandwich plate in detail. It was found that the deflection of the FGM plate is greater than that of the FGM sandwich plate, while the normal stress of the FGM plate is smaller than that of the FGM sandwich plate. Moreover, the FGM plate and FGM sandwich plate are sensitive to nonlinear temperature changes.

## 1. Introduction

The sandwich structure [1,2,3] is composed of two thin and hard faceplates and a light and thick core layer and is a widely used composite material structure in the composite material industry. Because of its excellent material properties, it is widely used in various engineering structures in aerospace, shipbuilding, residential buildings, and so on. Traditional sandwich structures use adhesives to bond two faceplates and the core layer together; there are differences in the material properties and temperature coefficients between different materials, resulting in significant interlayer stress at the interface between the core layer and the faceplate, ultimately leading to delamination in the sandwich structure. In order to solve the problems of sandwich structures, functionally graded materials have been applied to these structures. Functionally graded materials [4,5,6] are advanced non-uniform composite materials composed of different phases, and their material properties will continuously change in one or more directions. Therefore, functionally graded materials are used to alleviate interface problems in sandwich structures and achieve their applications under specific requirements.

In recent years, because of the widespread application of FGM in sandwich structures, a large number of researchers have conducted research on the mechanical properties of functionally graded material structures and functionally graded material sandwich structures. Before conducting research on FGM sandwich structures, it is necessary to have a deep understanding of FGM structures. He et al. [7] proposed a finite element formulation based on classical laminated plate theory to analyze the shape and vibration control of functionally graded material plates integrated with piezoelectric sensors and actuators. Yang et al. [8] studied the vibration and acoustic amplitude characteristics of FGM plates in a thermal environment and found that material distribution and temperature have a significant impact on the vibration and acoustic response of FGM plates. Wang et al. [9] studied the vibration behavior of longitudinally moving porous functionally graded material plates by using the d’Alembert principle and Galerkin method. Hien and Noh [10] conducted a study on the free vibration of functionally graded plates with spatially varying material properties based on random geometric analysis and found that the randomness of material properties and the correlation between elastic modulus and mass density have a significant impact on the response. Zhou et al. [11] proposed a unified solution considering both classical and non-classical boundary conditions to analyze the vibration and flutter behavior of supersonic porous functionally graded material plates. Chen et al. [12] studied the nonlinear vibration of a bidirectional functionally graded material plate with geometric defects under transverse harmonic excitation and found that changes in gradient parameters and the presence of geometric defects did indeed alter the nonlinearity of the resonance response. Yin et al. [13] combined three-dimensional elastic theory with the finite element method to study the bending and free vibration of porous functionally graded material plates. Nguyen [14] combined Navier’s solution with the finite element method to analyze the mechanical properties of double-layer FGM plates with metal shear connections on elastic foundations.

After understanding some of the mechanical properties of FGM structures, researchers conducted further research on the mechanical properties of FGM sandwich structures based on these mechanical properties. Naveenkumar et al. [15] studied the analytical formulations and solutions for the flexural analysis of functionally graded material sandwich plates with available high-order fine-grained computational models and presented numerous numerical results for in-plane, transverse displacements, and stresses. Liu et al. [16] studied the nonlinear dynamic response of porous functionally graded sandwich cylindrical shells embedded in elastic media by using modified Donnell nonlinear shell theory and Hamilton’s principle. Hirane et al. [17] proposed a fixed C_0_ high-order layered finite element model to analyze the static and free vibration of FGM sandwich plates under different boundary conditions. Naghavi et al. [18] combined refined plate theory with the finite strip method to perform mechanical bending analysis on two functionally graded sandwich plates under different boundary conditions. Vinh [19] combined high-order shear deformation theory with the finite element method for the study of bidirectional functionally graded sandwich plates. Finally, the parameters were studied. Liu et al. [20] used shear deformation shell theory and Hamilton’s principle to study the impact response of a sandwich cylindrical shell composed of a porous functionally graded core. In terms of thermal environment, researchers have also conducted extensive research. Zenkour [21] used shear deformation plate theory and classical plate theory to study the bending response of asymmetric functionally graded material sandwich plates under thermo-mechanical loads, respectively. Pandey and Pradyumna [22] studied the thermally induced vibration of functionally graded sandwich plates and shells using a finite element method based on higher-order delamination theory and found that this method made it easy to study FGM sandwich plates and shells under rapid heating. Zarga et al. [23] used a simple quasi-3D shear deformation theory to perform a thermal bending analysis of functionally graded material sandwich structures, and this theory considered a new kind of kinematics. Trinh and Kim [24] studied the nonlinear stability of intermediate thickness functionally graded material sandwich shells under thermal mechanical loads on elastic foundations based on first-order shear deformation theory. Joseph and Mohnty [25] conducted a free vibration and parametric instability analysis of a three-layer sandwich plate with a viscoelastic core layer and a functionally graded material constraint layer using a finite element method. Bouamoud et al. [26] used a four-variable plate model to study the bending behavior of two FGM sandwich plates under thermo-mechanical load, and this model only involved four unknowns. Finally, they carried out a detailed parameter study. Yoosefian et al. [27] employed first-order shear theory and Van Karman’s nonlinear strain–displacement relationship to investigate the nonlinear thermo-mechanical bending of circular/ring functionally graded material sandwich plates. Trinh et al. [28] studied the deterministic and stochastic dynamics of four functionally graded sandwich plates without a layered structure under thermo-mechanical loads based on the third-order shear deformation theory and found that the presence of thermal loads made the random response of the plates more diverse. Daikh et al. [29] applied the high-order shear deformation theory to study the thermo-mechanical bending behavior of functionally graded sandwich plates and carried out parameter analysis. Saffari et al. [30] studied the sound transmission loss of an inflatable rectangular double-walled sandwich smart magneto-electro-elastic plate with a porous FGM core under the external average airflow under different temperature distributions and carried out a parametric study on it. Belkhodja et al. [31] analyzed the buckling and bending response of simply supported FGM sandwich plates under thermal load using new quasi-three-dimensional and two-dimensional high-order shear deformation theories. Kiarasi et al. [32] studied the buckling response of sandwich plates reinforced by carbon nanotubes with a polymer core layer and two faceplates with graded material properties in a thermal environment.

Scholars have studied the mechanical properties of functionally graded material plate and sandwich structures, including their vibration, bending, and stability under various conditions. However, there is not much research on the bending of the FGM plate and FGM sandwich plate under thermal mechanical loads. In order to fill this gap, this paper studies the bending behavior of symmetric functionally graded material plate and functionally graded material sandwich plate under thermo-mechanical load. In order to analyze this behavior, a new thermo-mechanical bending model was established that can analyze both the FGM plate and FGM sandwich plate. Based on the theory of refined shear deformation and the principle of minimum potential energy, four control equations were established, which had only four positional variables. Among them, for the temperature field, a nonlinear form that varied along the thickness direction was adopted, which was based on three displacement fields: classical plate theory, first-order shear deformation theory, and high-order shear deformation theory. Next, in order to solve the control equation, the Navier method was used to obtain analytical solutions for the FGM plate and FGM sandwich plate under simply supported boundary conditions. During this process, three different forms were used for the shape function of the displacement field. We compared the thermodynamic results of the FGM plate obtained with the results in the literature to verify the accuracy of the model proposed in this paper. Finally, parameter studies were conducted on the symmetric functionally graded material plate and functionally graded material sandwich plate, and the effects of volume fraction index, side-to-thickness ratio, nonlinear temperature, and core layer thickness on the thermo-mechanical bending performance of the symmetric functionally graded material plate and functionally graded material sandwich plate under simply supported boundary conditions were analyzed in detail.

## 2. Theoretical Models and Formulas

**Figure 1:**
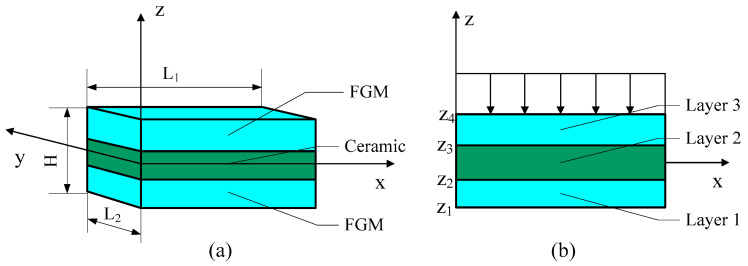
Geometry of the FGM sandwich rectangular plate in Cartesian coordinates. (**a**) three dimensional coordinates of FGM sandwich plate; (**b**) two dimensional coordinates of FGM sandwich plate.

In this paper, the FGM sandwich plate is composed of two FGM faceplates and a homogeneous material core layer. The core layer is a ceramic layer. The length, width, and thickness of the FGM sandwich plate are $L_{1}$, $L_{2}$, and H, respectively. The established coordinate system is shown in Figure 1. There is a transverse load q on the top surface of the FGM sandwich plate.

The material properties of FGM can be expressed by the Voigt model as [33]:

(1) $$p\left(z\right)=p_{c}V_{c}\left(z\right)+p_{m}V_{m}\left(z\right)\;$$

where $p_{c}$ and $p_{m}$ are the material properties (such as Young’s modulus, Poisson’s ratio, thermal expansion coefficient, etc.) of ceramics and metals, respectively. $V_{c}$ and $V_{m}$ are the volume fractions of ceramic and metal, respectively, and they satisfy the relationship of $V_{c}+V_{m}=1$.

In the FGM sandwich plate, $V_{c}^{\left(i\right)}$ is expressed as:

(2) $$\{\begin{array}{c} V_{c}^{\left(1\right)}\left(z\right)={\left(\frac{z\;-\;z_{1}}{z_{2}\;-\;z_{1}}\right)}^{s}\;\;z\in \left[z_{1},z_{2}\right]\\ V_{c}^{\left(2\right)}\left(z\right)=1\;\;\;\;\;z\in \left[z_{2},z_{3}\right]\\ V_{c}^{\left(3\right)}\left(z\right)={\left(\frac{z\;-\;z_{4}}{z_{3}\;-\;z_{4}}\right)}^{s}\;\;z\in \left[z_{3},z_{4}\right] \end{array}$$

where s is volume fraction index, while s=0 represents a fully ceramic plate.

According to the refined shear deformation theory, the following displacement field can be obtained [34]:

(3) $$\left\{ \begin{array}{l} \tilde{u}\left(x,y,z\right)={\tilde{u}}_{1}\left(x,y\right)-z\frac{\partial {\tilde{w}}_{1}}{\partial x}-k\left(z\right)\frac{\partial {\tilde{w}}_{2}}{\partial x}\\ \tilde{v}\left(x,y,z\right)={\tilde{v}}_{1}\left(x,y\right)-z\frac{\partial {\tilde{w}}_{1}}{\partial y}-k\left(z\right)\frac{\partial {\tilde{w}}_{2}}{\partial y}\\ \tilde{w}\left(x,y,z\right)={\tilde{w}}_{1}\left(x,y\right)+{\tilde{w}}_{2}\left(x,y\right) \end{array}\right.$$

where ${\tilde{u}}_{1}$ and ${\tilde{v}}_{1}$ are the tensile parts in the *x* and *y* directions, respectively. ${\tilde{w}}_{1}$ and ${\tilde{w}}_{2}$ are bending component and shearing component, respectively. k(z) is the shape function of z, and k(z)=z−ζ(z). ζ(z) adopts the shape function form proposed by Reissner, Reddy, and Touratier [35,36,37]. They can be given by:

(4) $$\mathrm{Reissner}:\zeta \left(z\right)=\frac{5z}{4}\left(1-\frac{4z^{2}}{3H^{2}}\right),\mathrm{Reddy}:\zeta \left(z\right)=z\left(1-\frac{4z^{2}}{3H^{2}}\right),\mathrm{Touratier}:\zeta \left(z\right)=\frac{H}{\pi }\sin\left(\frac{\pi z}{H}\right)$$

The shape function in this work defaults to the shape function proposed by Reissner.

The relationship between strain and displacement field are given by:

(5) $${\tilde{\varepsilon }}_{xx}=\frac{\partial \tilde{u}}{\partial x},{\tilde{\varepsilon }}_{yy}=\frac{\partial \tilde{v}}{\partial y},{\tilde{\varepsilon }}_{zz}=\frac{\partial \tilde{w}}{\partial z},{\tilde{\gamma }}_{xy}=\frac{\partial \tilde{v}}{\partial x}+\frac{\partial \tilde{u}}{\partial y},{\tilde{\gamma }}_{yz}=\frac{\partial \tilde{w}}{\partial y}+\frac{\partial \tilde{v}}{\partial z},{\tilde{\gamma }}_{xz}=\frac{\partial \tilde{w}}{\partial x}+\frac{\partial \tilde{u}}{\partial z}$$

Substituting Equation (3) into Equation (5) gives:

(6) $$\left(\begin{array}{c} {\tilde{\varepsilon }}_{xx}\\ {\tilde{\varepsilon }}_{yy}\\ {\tilde{\gamma }}_{xy} \end{array}\right)=\left(\begin{array}{c} {\tilde{\varepsilon }}_{xx}^{1}\\ {\tilde{\varepsilon }}_{yy}^{1}\\ {\tilde{\gamma }}_{xy}^{1} \end{array}\right)+z\left(\begin{array}{c} {\tilde{\kappa }}_{xx}^{1}\\ {\tilde{\kappa }}_{yy}^{1}\\ {\tilde{\kappa }}_{xy}^{1} \end{array}\right)+k\left(z\right)\left(\begin{array}{c} {\tilde{\kappa }}_{xx}^{2}\\ {\tilde{\kappa }}_{yy}^{2}\\ {\tilde{\kappa }}_{xy}^{2} \end{array}\right),{\tilde{\varepsilon }}_{zz}=0,\left(\begin{array}{c} {\tilde{\gamma }}_{xz}\\ {\tilde{\gamma }}_{yz} \end{array}\right)=\left[1-k^{\prime }\left(z\right)\right]\left(\begin{array}{c} {\tilde{\gamma }}_{xz}^{2}\\ {\tilde{\gamma }}_{yz}^{2} \end{array}\right)$$

where

(7) $$\left(\begin{array}{c} {\tilde{\varepsilon }}_{xx}^{1}\\ {\tilde{\varepsilon }}_{yy}^{1}\\ {\tilde{\gamma }}_{xy}^{1} \end{array}\right)=\left(\begin{array}{c} \frac{\partial {\tilde{u}}_{1}}{\partial x}\\ \frac{\partial {\tilde{v}}_{1}}{\partial y}\\ \frac{\partial {\tilde{u}}_{1}}{\partial y}+\frac{\partial {\tilde{v}}_{1}}{\partial x} \end{array}\right)\left(\begin{array}{c} {\tilde{\kappa }}_{xx}^{1}\\ {\tilde{\kappa }}_{yy}^{1}\\ {\tilde{\kappa }}_{xy}^{1} \end{array}\right)=-\left(\begin{array}{c} \frac{\partial^{2}{\tilde{w}}_{1}}{\partial x^{2}}\\ \frac{\partial^{2}{\tilde{w}}_{1}}{\partial y^{2}}\\ 2\frac{\partial^{2}{\tilde{w}}_{1}}{\partial x\partial y} \end{array}\right)\left(\begin{array}{c} {\tilde{\kappa }}_{xx}^{2}\\ {\tilde{\kappa }}_{yy}^{2}\\ {\tilde{\kappa }}_{xy}^{2} \end{array}\right)=-\left(\begin{array}{c} \frac{\partial^{2}{\tilde{w}}_{2}}{\partial x^{2}}\\ \frac{\partial^{2}{\tilde{w}}_{2}}{\partial y^{2}}\\ 2\frac{\partial^{2}{\tilde{w}}_{2}}{\partial x\partial y} \end{array}\right)\left(\begin{array}{c} {\tilde{\gamma }}_{xz}^{2}\\ {\tilde{\gamma }}_{yz}^{2} \end{array}\right)=\left(\begin{array}{c} \frac{\partial {\tilde{w}}_{2}}{\partial x}\\ \frac{\partial {\tilde{w}}_{2}}{\partial y} \end{array}\right)$$

According to the above strain field, the stress field of the FGM sandwich plate can be obtained by using the constitutive relationship [21]:

(8) $${\left(\begin{array}{c} {\tilde{\sigma }}_{xx}\\ {\tilde{\sigma }}_{yy}\\ {\tilde{\tau }}_{yz}\\ {\tilde{\tau }}_{xz}\\ {\tilde{\tau }}_{xy} \end{array}\right)}^{\left(i\right)}={\left[\begin{array}{ccccc} {\tilde{S}}_{11} & {\tilde{S}}_{12} & 0 & 0 & 0\\ {\tilde{S}}_{12} & {\tilde{S}}_{22} & 0 & 0 & 0\\ 0 & 0 & {\tilde{S}}_{44} & 0 & 0\\ 0 & 0 & 0 & {\tilde{S}}_{55} & 0\\ 0 & 0 & 0 & 0 & {\tilde{S}}_{66} \end{array}\right]}^{\left(i\right)}{\left(\begin{array}{c} {\tilde{\varepsilon }}_{xx}-\alpha \tilde{T}\\ {\tilde{\varepsilon }}_{yy}-\alpha \tilde{T}\\ {\tilde{\gamma }}_{yz}\\ {\tilde{\gamma }}_{xz}\\ {\tilde{\gamma }}_{xy} \end{array}\right)}^{\left(i\right)}\left(i=1,2,3\right)$$

where ${\tilde{S}}_{11},{\tilde{S}}_{12},{\tilde{S}}_{22},{\tilde{S}}_{44},{\tilde{S}}_{55},{\tilde{S}}_{66}$ can be expressed as:

(9) $${\tilde{S}}_{11}^{\left(i\right)}={\tilde{S}}_{22}^{\left(i\right)}=\frac{E^{\left(i\right)}\left(z\right)}{1-{\left(\vartheta^{\left(i\right)}\right)}^{2}},{\tilde{S}}_{12}^{\left(i\right)}=\vartheta^{\left(i\right)}{\tilde{S}}_{11}^{\left(i\right)},{\tilde{S}}_{44}^{\left(i\right)}={\tilde{S}}_{55}^{\left(i\right)}={\tilde{S}}_{66}^{\left(i\right)}=\frac{E^{\left(i\right)}\left(z\right)}{2\left(1+\vartheta^{\left(i\right)}\right)}$$

where $E^{\left(i\right)}$ and $\vartheta^{\left(i\right)}$ are the Young’s modulus and Poisson’s ratio of layer i, respectively.

The total strain potential energy of the FGM sandwich plate is [38]:

(10) $${\tilde{U}}_{p}=\frac{1}{2}\int_{V}\left[{\tilde{\sigma }}_{xx}^{\left(i\right)}{\left({\tilde{\varepsilon }}_{xx}-\alpha \tilde{T}\right)}^{\left(i\right)}+{\tilde{\sigma }}_{yy}^{\left(i\right)}{\left({\tilde{\varepsilon }}_{yy}-\alpha \tilde{T}\right)}^{\left(i\right)}+{\tilde{\tau }}_{xy}^{\left(i\right)}{\tilde{\gamma }}_{xy}^{\left(i\right)}+{\tilde{\tau }}_{xz}^{\left(i\right)}{\tilde{\gamma }}_{xz}^{\left(i\right)}+{\tilde{\tau }}_{yz}^{\left(i\right)}{\tilde{\gamma }}_{yz}^{\left(i\right)}\right]dV,\;\left(i=1,2,3\right)$$

where $\alpha^{\left(i\right)}$ means the thermal expansion coefficient of layer i, and *V* is the volume of the FGM sandwich plate.

The external force is defined by:

(11) $$\tilde{W}=\int_{\Omega }q\tilde{w}d\Omega$$

where Ω is the top surface of the FGM sandwich plate.

The variational forms of Equations (10) and (11) are expressed as:

(12) $${\delta \tilde{U}}_{p}=\int_{V}\left[{\tilde{\sigma }}_{xx}^{\left(i\right)}\delta {\tilde{\varepsilon }}_{xx}^{\left(i\right)}+{\tilde{\sigma }}_{yy}^{\left(i\right)}\delta {\tilde{\varepsilon }}_{yy}^{\left(i\right)}+{\tilde{\tau }}_{xy}^{\left(i\right)}\delta {\tilde{\gamma }}_{xy}^{\left(i\right)}+{\tilde{\tau }}_{xz}^{\left(i\right)}\delta {\tilde{\gamma }}_{xz}^{\left(i\right)}+{\tilde{\tau }}_{yz}^{\left(i\right)}\delta {\tilde{\gamma }}_{yz}^{\left(i\right)}\right]dV\left(i=1,2,3\right),\delta \tilde{W}=\int_{\Omega }q\delta \tilde{w}d\Omega$$

According to the principle of minimum potential energy, one gets:

(13) $$\int_{V}\left[{\tilde{\sigma }}_{xx}^{\left(i\right)}\delta {\tilde{\varepsilon }}_{xx}^{\left(i\right)}+{\tilde{\sigma }}_{yy}^{\left(i\right)}\delta {\tilde{\varepsilon }}_{yy}^{\left(i\right)}+{\tilde{\tau }}_{xy}^{\left(i\right)}\delta {\tilde{\gamma }}_{xy}^{\left(i\right)}+{\tilde{\tau }}_{xz}^{\left(i\right)}\delta {\tilde{\gamma }}_{xz}^{\left(i\right)}+{\tilde{\tau }}_{yz}^{\left(i\right)}\delta {\tilde{\gamma }}_{yz}^{\left(i\right)}\right]dV-\int_{\Omega }q\delta \tilde{w}d\Omega =0$$

Substituting Equations (6) and (8) into Equation (13) and integrating *z*, Equation (13) can be rewritten as:

(14) $$\begin{array}{l} \int_{\Omega }[{\tilde{N}}_{xx}\delta {\tilde{\varepsilon }}_{xx}^{1}+{\tilde{N}}_{yy}\delta {\tilde{\varepsilon }}_{yy}^{1}+{\tilde{N}}_{xy}\delta {\tilde{\gamma }}_{xy}^{1}+{\tilde{M}}_{xx}^{1}\delta {\tilde{\kappa }}_{xx}^{1}+{\tilde{M}}_{yy}^{1}\delta {\tilde{\kappa }}_{yy}^{1}+{\tilde{M}}_{xy}^{1}\delta {\tilde{\kappa }}_{xy}^{1}+{\tilde{M}}_{xx}^{2}\delta {\tilde{\kappa }}_{xx}^{2}\\ +{\tilde{M}}_{yy}^{2}\delta {\tilde{\kappa }}_{yy}^{2}+{\tilde{M}}_{xy}^{2}\delta {\tilde{\kappa }}_{xy}^{2}+{\tilde{Q}}_{xz}^{2}\delta {\tilde{\kappa }}_{xz}^{2}+{\tilde{Q}}_{yz}^{2}\delta {\tilde{\kappa }}_{yz}^{2}]d\Omega -\int_{\Omega }q\delta \tilde{w}d\Omega =0 \end{array}$$

where ${\tilde{N}}_{xx},{\tilde{N}}_{yy},{\tilde{N}}_{xy},{\tilde{M}}_{{}_{xx}}^{1},{\tilde{M}}_{yy}^{1},{\tilde{M}}_{{}_{xy}}^{1},{\tilde{M}}_{{}_{xx}}^{2},{\tilde{M}}_{{}_{yy}}^{2},{\tilde{M}}_{{}_{xy}}^{2},{\tilde{Q}}_{{}_{xz}}^{2},{\tilde{Q}}_{{}_{yz}}^{2}$ can be given by:

(15) $$\left[\begin{array}{ccc} {\tilde{N}}_{xx} & {\tilde{N}}_{yy} & {\tilde{N}}_{xy}\\ {\tilde{M}}_{xx}^{1} & {\tilde{M}}_{yy}^{1} & {\tilde{M}}_{xy}^{1}\\ {\tilde{M}}_{xx}^{2} & {\tilde{M}}_{yy}^{2} & {\tilde{M}}_{xy}^{2} \end{array}\right]=\overset{3}{\underset{i=1}{\sum }}\int_{z_{i}}^{z_{i+1}}\left(\begin{array}{c} 1\\ z\\ k\left(z\right) \end{array}\right){\left(\begin{array}{ccc} {\tilde{\sigma }}_{xx} & {\tilde{\sigma }}_{yy} & {\tilde{\sigma }}_{xy} \end{array}\right)}^{\left(i\right)}dz,\left[\begin{array}{c} {\tilde{Q}}_{xz}^{2}\\ {\tilde{Q}}_{yz}^{2} \end{array}\right]=\overset{3}{\underset{i=1}{\sum }}\int_{z_{i}}^{z_{i+1}}\left[1-k^{\prime }\left(z\right)\right]{\left(\begin{array}{c} {\tilde{\tau }}_{xz}\\ {\tilde{\tau }}_{yz} \end{array}\right)}^{\left(i\right)}dz$$

Substituting Equation (7) into Equation (14) and integrating by parts, then let the coefficients before $\delta {\tilde{u}}_{1}$, $\delta {\tilde{v}}_{1}$, $\delta {\tilde{w}}_{1}$, and $\delta {\tilde{w}}_{2}$ be zero, and the following differential equation can be obtained as:

(16) $$\begin{array}{l} \delta {\tilde{u}}_{1}:\frac{\partial {\tilde{N}}_{xx}}{\partial x}+\frac{\partial {\tilde{N}}_{xy}}{\partial y}=0,\delta {\tilde{v}}_{1}:\frac{\partial {\tilde{N}}_{xy}}{\partial x}+\frac{\partial {\tilde{N}}_{yy}}{\partial y}=0,\delta {\tilde{w}}_{1}:\frac{\partial^{2}{\tilde{M}}_{xx}^{1}}{\partial x^{2}}+2\frac{\partial^{2}{\tilde{M}}_{xy}^{1}}{\partial x\partial y}+\frac{\partial^{2}{\tilde{M}}_{yy}^{1}}{\partial y^{2}}+q=0\\ \;\delta {\tilde{w}}_{2}:\frac{\partial^{2}{\tilde{M}}_{xx}^{2}}{\partial x^{2}}+2\frac{\partial^{2}{\tilde{M}}_{xy}^{2}}{\partial x\partial y}+\frac{\partial^{2}{\tilde{M}}_{yy}^{2}}{\partial y^{2}}+\frac{\partial {\tilde{Q}}_{xz}^{2}}{\partial x}+\frac{\partial {\tilde{Q}}_{yz}^{2}}{\partial y}+q=0 \end{array}$$

Substituting Equations (6) and (8) into Equation (15) gives:

(17) $$\left(\begin{array}{c} \tilde{N}\\ {\tilde{M}}^{1}\\ {\tilde{M}}^{2} \end{array}\right)=\left[\begin{array}{ccc} \tilde{A} & {\tilde{A}}^{s} & {\tilde{B}}^{s}\\ A^{s} & \tilde{B} & {\tilde{C}}^{s}\\ {\tilde{B}}^{s} & {\tilde{C}}^{s} & \tilde{C} \end{array}\right]\left(\begin{array}{c} {\tilde{\varepsilon }}^{1}\\ {\tilde{\kappa }}^{1}\\ {\tilde{\kappa }}^{2} \end{array}\right)-\left(\begin{array}{c} {\tilde{N}}^{t}\\ {\tilde{M}}^{1t}\\ {\tilde{M}}^{2t} \end{array}\right),\left[\begin{array}{c} {\tilde{Q}}_{yz}^{2}\\ {\tilde{Q}}_{xz}^{2} \end{array}\right]=\left[\begin{array}{cc} {\tilde{E}}_{44} & 0\\ 0 & {\tilde{E}}_{55} \end{array}\right]\left(\begin{array}{c} {\tilde{\gamma }}_{yz}^{2}\\ {\tilde{\gamma }}_{xz}^{2} \end{array}\right)$$

where

(18) $$\begin{array}{l} {\left(\tilde{N},{\tilde{M}}^{1},{\tilde{M}}^{2}\right)}^{T}=({\tilde{N}}_{xx},{\tilde{N}}_{yy},{\tilde{N}}_{xy},{\tilde{M}}_{xx}^{1},{\tilde{M}}_{yy}^{1},{\tilde{M}}_{xy}^{1},{\tilde{M}}_{xx}^{2},{\tilde{M}}_{yy}^{2},{\tilde{M}}_{xy}^{2})\\ {\left({\tilde{N}}^{t},{\tilde{M}}^{1t},{\tilde{M}}^{2t}\right)}^{T}={\left({\tilde{N}}_{xx}^{t},{\tilde{N}}_{yy}^{t},0,{\tilde{M}}_{xx}^{1t},{\tilde{M}}_{yy}^{1t},0,{\tilde{M}}_{xx}^{2t},{\tilde{M}}_{yy}^{2t},0\right)}^{T}\\ {\left({\tilde{\varepsilon }}^{1},{\tilde{\kappa }}^{1},{\tilde{\kappa }}^{2}\right)}^{T}=({\tilde{\varepsilon }}_{xx}^{1},{\tilde{\varepsilon }}_{yy}^{1},{\tilde{\gamma }}_{xy}^{1},{\tilde{\kappa }}_{xx}^{1},{\tilde{\kappa }}_{yy}^{1},{\tilde{\kappa }}_{xy}^{1},{\tilde{\kappa }}_{xx}^{2},{\tilde{\kappa }}_{yy}^{2},{\tilde{\kappa }}_{xy}^{2}{)}^{T} \end{array}$$

and

(19) $$\begin{array}{l} \tilde{A}=\left(\begin{array}{ccc} {\tilde{A}}_{11} & {\tilde{A}}_{12} & 0\\ {\tilde{A}}_{12} & {\tilde{A}}_{22} & 0\\ 0 & 0 & {\tilde{A}}_{66} \end{array}\right){\tilde{A}}^{1}=\left(\begin{array}{ccc} {\tilde{A}}_{11}^{s} & {\tilde{A}}_{12}^{s} & 0\\ {\tilde{A}}_{12}^{s} & {\tilde{A}}_{22}^{s} & 0\\ 0 & 0 & {\tilde{A}}_{66}^{s} \end{array}\right)\tilde{B}=\left(\begin{array}{ccc} {\tilde{B}}_{11} & {\tilde{B}}_{12} & 0\\ {\tilde{B}}_{12} & {\tilde{B}}_{22} & 0\\ 0 & 0 & {\tilde{B}}_{66} \end{array}\right)\\ {\tilde{B}}^{s}=\left(\begin{array}{ccc} {\tilde{B}}_{11}^{s} & {\tilde{B}}_{12}^{s} & 0\\ {\tilde{B}}_{12}^{s} & {\tilde{B}}_{22}^{s} & 0\\ 0 & 0 & {\tilde{B}}_{66}^{s} \end{array}\right)\tilde{C}=\left(\begin{array}{ccc} {\tilde{C}}_{11} & {\tilde{C}}_{12} & 0\\ {\tilde{C}}_{12} & {\tilde{C}}_{22} & 0\\ 0 & 0 & {\tilde{C}}_{66} \end{array}\right){\tilde{C}}^{s}=\left(\begin{array}{ccc} {\tilde{C}}_{11}^{s} & {\tilde{C}}_{12}^{s} & 0\\ {\tilde{C}}_{12}^{s} & {\tilde{C}}_{22}^{s} & 0\\ 0 & 0 & {\tilde{C}}_{66}^{s} \end{array}\right) \end{array}$$

The specific forms of elements in matrices $\tilde{A},{\tilde{A}}^{s},\tilde{B},{\tilde{B}}^{s},\tilde{C},{\tilde{C}}^{s}$ and ${\tilde{E}}_{44},{\tilde{E}}_{55}$ can be written as:

(20) $$\begin{array}{l} \left(\begin{array}{c} {\tilde{A}}_{11}\\ {\tilde{A}}_{12}\\ {\tilde{A}}_{66} \end{array}\begin{array}{c} {\tilde{A}}_{11}^{s}\\ {\tilde{A}}_{12}^{s}\\ {\tilde{A}}_{66}^{s} \end{array}\begin{array}{c} {\tilde{B}}_{11}\\ {\tilde{B}}_{12}\\ {\tilde{B}}_{66} \end{array}\begin{array}{c} {\tilde{B}}_{11}^{s}\\ {\tilde{B}}_{12}^{s}\\ {\tilde{B}}_{66}^{s} \end{array}\begin{array}{c} {\tilde{C}}_{11}\\ {\tilde{C}}_{12}\\ {\tilde{C}}_{66} \end{array}\begin{array}{c} {\tilde{C}}_{11}^{s}\\ {\tilde{C}}_{12}^{s}\\ {\tilde{C}}_{66}^{s} \end{array}\right)=\overset{3}{\underset{i=1}{\sum }}\int_{z_{i}}^{z_{i+1}}\left(\begin{array}{c} {\tilde{S}}_{11}^{\left(i\right)}\\ {\tilde{S}}_{12}^{\left(i\right)}\\ {\tilde{S}}_{66}^{\left(i\right)} \end{array}\right)(1\;\;\;\;z\;\;\;\;z^{2}\;\;\;\;k\left(z\right)\;\;\;\;zk\left(z\right)\;\;\;\;k^{2}\left(z\right))dz\\ \left(\begin{array}{c} {\tilde{E}}_{44}\\ {\tilde{E}}_{55} \end{array}\right)=\overset{3}{\underset{i=1}{\sum }}\int_{z_{i}}^{z_{i+1}}\left[1-k^{\prime }\left(z\right)\right]\left(\begin{array}{c} {\tilde{S}}_{44}^{\left(i\right)}\\ {\tilde{S}}_{55}^{\left(i\right)} \end{array}\right)dz \end{array}$$

The matrix elements related to thermal load in Equation (17) are ${\tilde{N}}_{xx}^{t},{\tilde{N}}_{yy}^{t},{\tilde{M}}_{xx}^{1t},{\tilde{M}}_{yy}^{1t},{\tilde{M}}_{xx}^{2t},{\tilde{M}}_{yy}^{2t}$. They can be written as:

(21) $$\left[\begin{array}{ccc} {\tilde{N}}_{xx}^{t} & {\tilde{M}}_{xx}^{1t} & {\tilde{M}}_{xx}^{2t}\\ {\tilde{N}}_{yy}^{t} & {\tilde{M}}_{yy}^{1t} & {\tilde{M}}_{yy}^{2t} \end{array}\right]=\overset{3}{\underset{i=1}{\sum }}\int_{z_{i}}^{z_{i+1}}{\left(\begin{array}{c} \left({\tilde{S}}_{11}+{\tilde{S}}_{12}\right)\alpha \tilde{T}\\ ({\tilde{S}}_{12}+{\tilde{S}}_{22})\alpha \tilde{T} \end{array}\right)}^{\left(i\right)}\left(\begin{array}{ccc} 1 & z & k\left(z\right) \end{array}\right)dz$$

For the temperature field *T*, the nonlinear temperature field that varies along the thickness of the plate used by Mantari [39] is adopted in the paper, and the specific form is as follows:

(22) $$\tilde{T}\left(x,y,z\right)={\tilde{T}}_{1}\left(x,y\right)+\frac{z}{H}{\tilde{T}}_{2}\left(x,y\right)+\frac{\zeta \left(z\right)}{H}{\tilde{T}}_{3}\left(x,y\right)$$

where ${\tilde{T}}_{1}\left(x,y\right)$ is the temperature field that does not change in the thickness direction, ${\tilde{T}}_{2}\left(x,y\right)$ is the temperature field that changes linearly, and ${\tilde{T}}_{3}\left(x,y\right)$ is the temperature field that changes nonlinearly.

Under the simply supported boundary condition, the following relations are obtained:

(23) $$\begin{array}{c} x=0,L_{1}:{\tilde{v}}_{1}={\tilde{w}}_{1}={\tilde{w}}_{2}=0,\frac{\partial {\tilde{w}}_{1}}{\partial y}=\frac{\partial {\tilde{w}}_{2}}{\partial y}=0,{\tilde{N}}_{xx}=0,{\tilde{M}}_{xx}^{1}={\tilde{M}}_{xx}^{2}=0\\ y=0,L_{2}:{\tilde{u}}_{1}={\tilde{w}}_{1}={\tilde{w}}_{2}=0,\frac{\partial {\tilde{w}}_{1}}{\partial x}=\frac{\partial {\tilde{w}}_{2}}{\partial x}=0,{\tilde{N}}_{yy}=0,{\tilde{M}}_{yy}^{1}={\tilde{M}}_{yy}^{2}=0 \end{array}$$

To solve the above model, the Navier method is used in this paper, and the following assumptions are made for bi-sinusoidal load, temperature field, and displacement field:

(24) $$q={\tilde{q}}_{0}\sin(\tilde{\lambda }x)\sin(\tilde{\varsigma }y),\left(\begin{array}{c} {\tilde{T}}_{1}\\ {\tilde{T}}_{2}\\ {\tilde{T}}_{3} \end{array}\right)=\left(\begin{array}{c} {\tilde{t}}_{1}\\ {\tilde{t}}_{2}\\ {\tilde{t}}_{3} \end{array}\right)\sin\left(\tilde{\lambda }x\right)\sin\left(\tilde{\varsigma }y\right),\left(\begin{array}{c} {\tilde{u}}_{1}\\ {\tilde{v}}_{1}\\ {\tilde{w}}_{1}\\ {\tilde{w}}_{2} \end{array}\right)=\left(\begin{array}{c} \tilde{U}\cos\left(\tilde{\lambda }x\right)\sin(\tilde{\varsigma }y)\\ \tilde{V}\sin\left(\tilde{\lambda }x\right)\cos\left(\tilde{\varsigma }y\right)\\ {\tilde{W}}_{1}\sin\left(\tilde{\lambda }x\right)\sin\left(\tilde{\varsigma }y\right)\\ {\tilde{W}}_{2}\sin\left(\tilde{\lambda }x\right)\sin\left(\tilde{\varsigma }y\right) \end{array}\right)$$

where ${\tilde{q}}_{0},{\tilde{t}}_{1},{\tilde{t}}_{2},{\tilde{t}}_{3},\tilde{U},\tilde{V},{\tilde{W}}_{1},{\tilde{W}}_{2}$ are constants. $\tilde{\lambda }=\pi /L_{1}$ and $\tilde{\varsigma }=\pi /L_{2}$.

Based on the above assumptions, the following operator equation can be obtained:

(25) $$\left[\tilde{\Gamma }\right]\left[\tilde{\Lambda }\right]=\left[\tilde{F}\right]$$

where $\left[\tilde{\Lambda }\right]={\left(\begin{array}{cccc} \tilde{U} & \tilde{V} & {\tilde{W}}_{1} & {\tilde{W}}_{2} \end{array}\right)}^{T}$, $\left[\tilde{\Gamma }\right]$ denotes the stiffness coefficient matrix, and $\left[\tilde{F}\right]$ denotes the displacement vector and the generalized force. For the stiffness coefficient matrix $\left[\tilde{\Gamma }\right]$ and the generalized force $\left[\tilde{F}\right]$ see Appendix A.

## 3. Model Validation and Numerical Analysis

In this part, a numerical example of the symmetric 1-0-1 FGM sandwich plate is given and discussed to verify the accuracy of the present method in predicting the bending of simply supported FGM sandwich plates under thermo-mechanical loads. In addition, several numerical examples of thermo-mechanical bending of symmetric FGM sandwich plates under simply supported boundary conditions are also given and analyzed.

The FGM is composed of titanium alloy (as metal) and zirconia (as ceramic). Their material properties [40] are listed in Table 1.

Unless mentioned otherwise, the following properties are used:

$$L_{1}/H=10,L_{1}=L_{2},{\tilde{q}}_{0}=100,{\tilde{t}}_{1}=0,{\tilde{t}}_{2}={\tilde{t}}_{3}=100K$$

The dimensionless deflection and stress are defined as [40]:

$$\begin{array}{l} \bar{\tilde{w}}=\frac{10^{3}}{{\tilde{q}}_{0}L_{1}^{4}/(E_{0}H^{3})+10^{3}\alpha_{0}{\tilde{t}}_{2}L_{1}^{2}/H}\tilde{w}\left(\frac{L_{1}}{2},\frac{L_{1}}{2}\right),{\bar{\tilde{\sigma }}}_{xx}=\frac{10^{3}}{q_{0}L_{1}^{2}/H^{2}+10E_{0}\alpha_{0}{\tilde{t}}_{2}L_{1}^{2}/H^{2}}{\tilde{\sigma }}_{xx}\left(\frac{L_{1}}{2},\frac{L_{2}}{2},\frac{H}{2}\right)\\ {\bar{\tilde{\tau }}}_{xz}=\frac{10^{3}}{q_{0}L_{1}/H+E_{0}\alpha_{0}{\tilde{t}}_{2}L_{1}/(10H)}{\tilde{\tau }}_{xz}\left(0,\frac{L_{2}}{2},0\right) \end{array}$$

where $E_{0}=1\mathrm{GPa},\;\alpha_{0}=10^{-6}/K$.

The sandwich structure is represented by the ratio of the thicknesses of each layer. This article uses five types of sandwich structures: 1-0-1, 1-3-1, 1-4-1, 1-5-1, and 1-6-1. For example, 1-0-1 represents $z_{1}=-H/2,z_{2}=0,z_{3}=0,z_{4}=H/2$, 1-3-1 represents $z_{1}=-H/2,z_{2}=-3H/10,z_{3}=3H/10,z_{4}=H/2$, and so on.

### 3.1. Symmetrical FGM Plate

In order to verify the accuracy of the model in this paper, the dimensionless deflections and stresses of 1-0-1 FGM sandwich plates (FGM plate) calculated according to the model in this paper under the simply supported condition were compared with the theoretical results of the sinusoidal shear deformation plate theory (SSDPT), the third-order shear deformation plate theory (TSDPT), and the first-order shear deformation plate theory (FSDPT). The results are shown in Table 2.

It can be seen from Table 2 that the differences between the results of this paper and those of the literature were within a certain range. For $\bar{\tilde{w}}$, the FSDPT theory was closer, for ${\bar{\tilde{\sigma }}}_{xx}$, the TSDPT theory was closer, and for ${\bar{\tilde{\tau }}}_{xz}$, all three theories were generally very close. Therefore, comparing the results of this paper with these three theories effectively verified the correctness of the proposed model. In addition, $\bar{\tilde{w}}$ of the FGM plate increased with the value of s, ${\bar{\tilde{\sigma }}}_{xx}$ of the FGM plate decreased with the increase in the value of s at s>0, and ${\bar{\tilde{\tau }}}_{xz}$ of the FGM plate fluctuated within a certain range at s>0.

### 3.2. Symmetrical FGM Sandwich Plates

Four symmetrical FGM sandwich plates (with layer thickness ratios 1-3-1, 1-4-1, 1-5-1, and 1-6-1) were selected for research here. Three shape functions called Reissner, Reddy, and Touratier were used to calculate the dimensionless center deflections $\bar{\tilde{w}}$, the normal stress ${\bar{\tilde{\sigma }}}_{xx}$, and the transverse shear stress ${\bar{\tilde{\tau }}}_{xz}$ under different volume fraction indices (s=0,1,3,5), respectively. The results are listed in Table 3, Table 4 and Table 5, respectively.

It can be seen from Table 3, Table 4 and Table 5 that $\bar{\tilde{w}}$ and ${\bar{\tilde{\sigma }}}_{xx}$ calculated by the Reissner shape function were the largest, and $\bar{\tilde{w}}$ and ${\bar{\tilde{\sigma }}}_{xx}$ calculated by the Touratier shape function were the smallest. When s>0, ${\bar{\tilde{\tau }}}_{xz}$ calculated by the Reddy shape function was the smallest, ${\bar{\tilde{\tau }}}_{xz}$ calculated by the Touratier shape function was the largest. $\bar{\tilde{w}}$ and ${\bar{\tilde{\tau }}}_{xz}$ of the FGM sandwich plates increased with the value of s for a given layer thickness ratio, while ${\bar{\tilde{\sigma }}}_{xx}$ decreased with the increase in the value of s at s>0. Moreover, when s>0, $\bar{\tilde{w}}$ of the symmetric FGM sandwich plates decreased with the increase in the core thickness, and ${\bar{\tilde{\sigma }}}_{xx}$ of the symmetric FGM sandwich plates increased with the core thickness.

### 3.3. Parameter Study

This section examines the effects of side-to-thickness ratio $L_{1}/H$, volume fraction index s, and nonlinear temperature ${\tilde{t}}_{3}$ on the deflection and stress of the different FGM sandwich plates that are studied.

**Figure 2:**
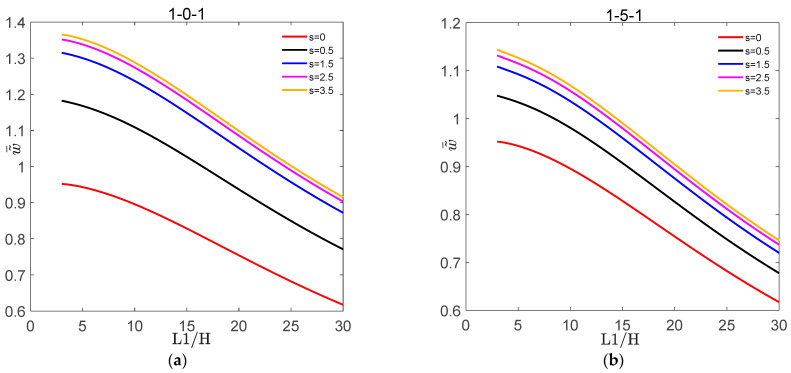
Variation of dimensionless deflection $\bar{\tilde{w}}$ with $L_{1}/H$ for the symmetric FGM plate and FGM sandwich plate under different volume fraction indices. (**a**) $\bar{\tilde{w}}$ of 1-0-1, (**b**) $\bar{\tilde{w}}$ of 1-5-1.

**Figure 3:**
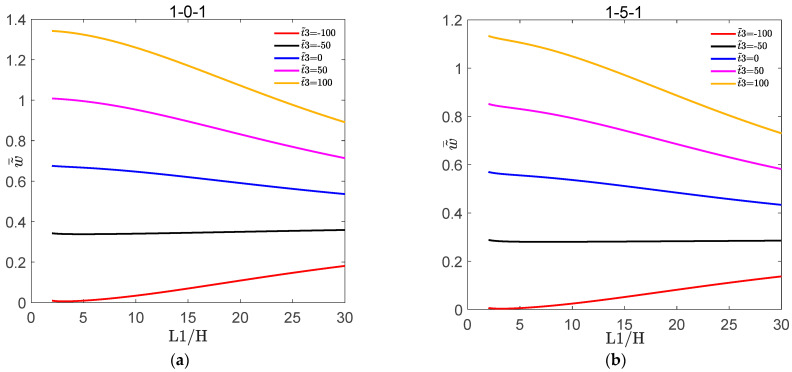
Variation of dimensionless deflection $\bar{\tilde{w}}$ with $L_{1}/H$ for the symmetric FGM plate and FGM sandwich plate under different nonlinear temperatures ${\tilde{t}}_{3}$. (**a**) $\bar{\tilde{w}}$ of 1-0-1; (**b**) $\bar{\tilde{w}}$ of 1-5-1.

**Figure 4:**
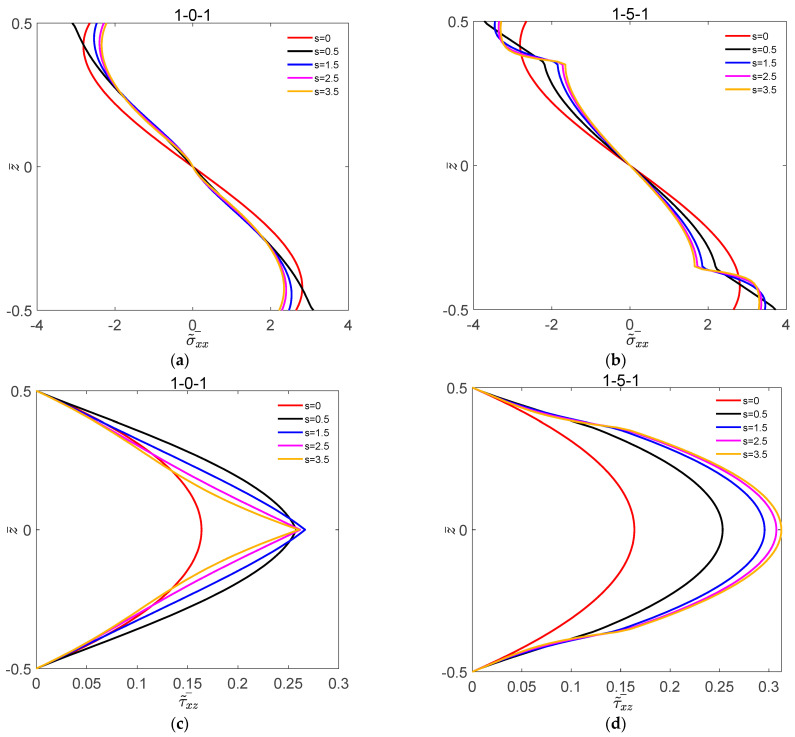
Variation of dimensionless stress ${\bar{\tilde{\sigma }}}_{xx}$ and ${\bar{\tilde{\tau }}}_{xz}$ with $\bar{z}$ for the symmetric FGM plate and FGM sandwich plate under different volume fraction indices s. (**a**) ${\bar{\tilde{\sigma }}}_{xx}$ of 1-0-1; (**b**) ${\bar{\tilde{\sigma }}}_{xx}$ of 1-5-1; (**c**) ${\bar{\tilde{\tau }}}_{xz}$ of 1-0-1; (**d**) ${\bar{\tilde{\tau }}}_{xz}$ of 1-5-1.

**Figure 5:**
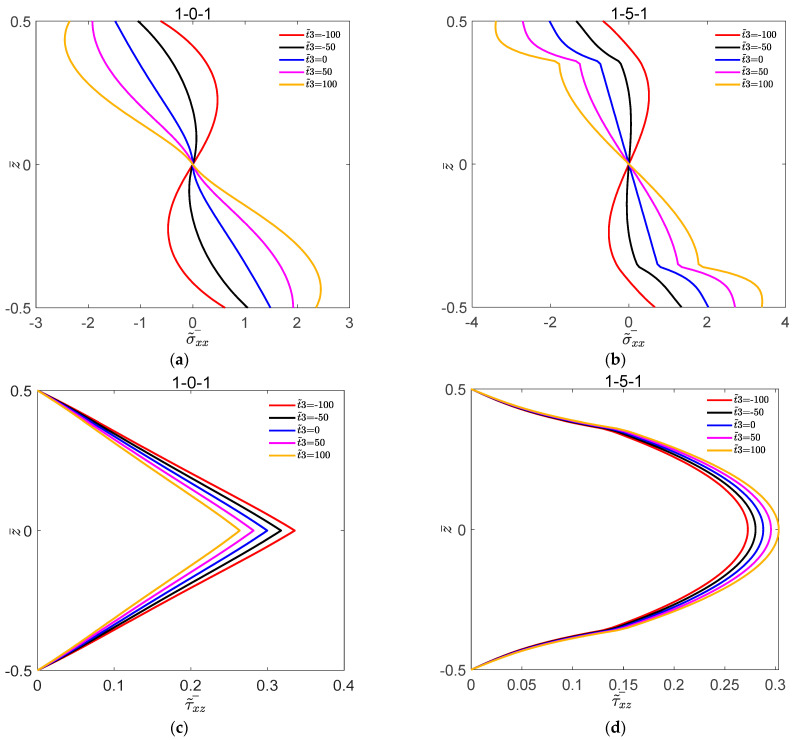
Variation of dimensionless stress ${\bar{\tilde{\sigma }}}_{xx}$ and ${\bar{\tilde{\tau }}}_{xz}$ with $\bar{z}$ for the symmetric FGM plate and FGM sandwich plate under different nonlinear temperatures ${\tilde{t}}_{3}$. (**a**) ${\bar{\tilde{\sigma }}}_{xx}$ of 1-0-1; (**b**) ${\bar{\tilde{\sigma }}}_{xx}$ of 1-5-1; (**c**) ${\bar{\tilde{\tau }}}_{xz}$ of 1-0-1; (**d**)${\bar{\tilde{\tau }}}_{xz}$ of 1-5-1.

Figure 2 shows the variation of dimensionless deflection $\bar{\tilde{w}}$ with $L_{1}/H$ for the symmetric FGM plate and FGM sandwich plate under different volume fraction indices s. Figure 3 shows the variation of dimensionless deflection $\bar{\tilde{w}}$ with $L_{1}/H$ for the symmetric FGM plate and FGM sandwich plate under different nonlinear temperatures ${\tilde{t}}_{3}$. Figure 4 shows the variation of ${\bar{\tilde{\sigma }}}_{xx}$ and ${\bar{\tilde{\tau }}}_{xz}$ with $\bar{z}$ for the symmetric FGM plate and FGM sandwich plate under different volume fraction indices s. Figure 5 shows the variation of ${\bar{\tilde{\sigma }}}_{xx}$ and ${\bar{\tilde{\tau }}}_{xz}$ with $\bar{z}$ for the symmetric FGM plate and FGM sandwich plate under different nonlinear temperatures ${\tilde{t}}_{3}$.

It can be seen from Figure 2 that $\bar{\tilde{w}}$ of the symmetric FGM plate and FGM sandwich plate decreased with the increase in $L_{1}/H$ for a given value of s. This was because, for the symmetric FGM plate and FGM sandwich plate with simply supported boundary conditions, the larger the side-to-thickness ratio, the greater the stiffness of the sandwich plate, resulting in a decrease in its deflection. The reason why $\bar{\tilde{w}}$ of the symmetric FGM plate was greater than that of the symmetric FGM sandwich plate was that the sandwich plate had an additional ceramic core layer compared to the single plate, which resulted in the stiffness of the FGM sandwich plate being greater than that of the FGM plate. In addition, $\bar{\tilde{w}}$ of the symmetric FGM plate and FGM sandwich plate increased with the value of s. This was because the ceramic volume content of the symmetric FGM plate and FGM sandwich plate decreased with the increase in volume fraction index s, resulting in a decrease in the stiffness of the plate.

It can be seen from Figure 3 that $\bar{\tilde{w}}$ of the symmetric FGM plate and FGM sandwich plate decreased with the increase in $L_{1}/H$ at ${\tilde{t}}_{3}\geq 0$. Meanwhile, $\bar{\tilde{w}}$ of the symmetric FGM plate and FGM sandwich plate increased with $L_{1}/H$ at ${\tilde{t}}_{3}<0$. It also can be seen from Figure 3 that $\bar{\tilde{w}}$ of the symmetric FGM plate and FGM sandwich plate increased with the value of ${\tilde{t}}_{3}$. This was because as the temperature increased, the stiffness of the symmetric FGM plate and FGM sandwich plate decreased.

For the sandwich plates with the top plate subjected to bi-sinusoidal loads, dimensionless positive stresses ${\bar{\tilde{\sigma }}}_{xx}$ are compressive stresses above the middle plane and tensile stresses below the middle plane.

It can be seen from Figure 4 that the stress was continuously distributed along the thickness direction. For a given value of s, the maximum compressive stress and the maximum tensile stress were on the upper and lower layers of the symmetric FGM plate and FGM sandwich plate, respectively. The maximum shear stress of the symmetric FGM plate and FGM sandwich plate occurred in the core of the plates. ${\bar{\tilde{\tau }}}_{xz}$ of the symmetric FGM sandwich plate increased with the value of s.

It can be seen from Figure 5 that the stress of the symmetric FGM plate and FGM sandwich plate were both sensitive to the value of ${\tilde{t}}_{3}$. Under the action of nonlinear temperature ${\tilde{t}}_{3}$, the stress of the FGM sandwich plate was greater than that of the FGM plate, and owing to the presence of nonlinear temperature ${\tilde{t}}_{3}$, the normal stress of the FGM sandwich plate will become less stable at the interface. For a given value of ${\tilde{t}}_{3}$, the maximum compressive stress and the maximum tensile stress were on the upper and lower layers of the symmetric FGM plate and FGM sandwich plate, respectively. The maximum shear stress of the symmetric FGM plate and FGM sandwich plate occurred in the core of the plates. ${\bar{\tilde{\tau }}}_{xz}$ of the symmetric FGM plate decreased with the increase in the value of ${\tilde{t}}_{3}$, and ${\bar{\tilde{\tau }}}_{xz}$ of the symmetric FGM sandwich plates was exactly the opposite.

## 4. Conclusions

In this study, the refined shear deformation theory was extended to the thermo-mechanical bending analysis of the symmetric FGM plate and FGM sandwich plate. Based on the principle of minimum potential energy, its governing equation was obtained. Its solution under simply supported boundary conditions was obtained by using the Navier method. To verify the accuracy of the theory presented in this paper, the bending results of the symmetric FGM plate under thermo-mechanical loading were compared with those in other studies. Finally, the effects of the volume fraction index, side-to-thickness ratio, core layer thickness, and nonlinear temperature on the deflection and stress of the symmetric FGM plate and FGM sandwich plates were investigated. The following conclusions were reached:

1. In this paper, there are only four control equations and unknown variables. The complexity and workload of calculation were significantly reduced.
2. By comparing the results of the symmetric 1-0-1 FGM sandwich plate with those published in the literature, it could be seen that the theoretical model in this paper was accurate in predicting the bending performance of FGM sandwich plates under thermo-mechanical load.
3. For the symmetric FGM plate and FGM sandwich plate, the stress was continuous, but not always smooth, especially at the interface.
4. Conclusion of the parameter study:
  (1) When the nonlinear temperature ${\tilde{t}}_{3}$ is constant, regardless of the volume fraction index s, the dimensionless deflection of the FGM plate and FGM sandwich plates will decrease with the increase in $L_{1}/H$. However, when the volume fraction index s is constant, and ${\tilde{t}}_{3}\geq 0$, the dimensionless deflection of the FGM plate and FGM sandwich plates will decrease with the increase in $L_{1}/H$, while when ${\tilde{t}}_{3}<0$, the dimensionless deflection of the FGM plate and FGM sandwich plates will increase with $L_{1}/H$.
  (2) For the symmetric FGM plate and FGM sandwich plate, the greater the volume fraction index s or the nonlinear temperature ${\tilde{t}}_{3}$, the greater the dimensionless deflection.
  (3) For the symmetric FGM plate and FGM sandwich plate, the maximum compressive stress is always generated on the top plate, and the maximum tensile stress is always generated on the bottom plate; the maximum shear stress of the symmetric FGM plate and FGM sandwich plate always occurs in the core of the plates.
  (4) When the layer thickness ratio and volume fraction index s are given, the dimensionless transverse shear stresses of the FGM plate will decrease with the increase in nonlinear temperature ${\tilde{t}}_{3}$, while the dimensionless transverse shear stresses of the symmetric FGM sandwich plate is exactly the opposite. In addition, the dimensionless normal stress of the FGM sandwich plate becomes unstable at the interface.
  (5) In any case, the dimensionless deflection of FGM plates is greater than that of FGM sandwich plates, and the dimensionless normal stress of FGM is smaller than that of FGM sandwich plates.

## Figures and Tables

**Table 1:** Material properties of metals and ceramics in FGM.

	Ti-6Al-4V	ZrO_2_
Young’s modulus (GPa)	66.2	117.0
Poisson’s ratio	1/3	1/3
Thermal expansion coefficient (10^−6^/K)	10.3	7.11

**Table 2:** Dimensionless deflections and stress of the FGM plate.

s	Theory	$\bar{\tilde{w}}$	${\bar{\tilde{\sigma }}}_{xx}$	${\bar{\tilde{\tau }}}_{xz}$
0	SSDPT [40]	0.796783	−2.388909	0.171603
	TSDPT [40]	0.808168	−2.461177	0.174481
	FSDPT [29]	0.895735	−3.597007	0.173624
	Present	0.895427	−2.650327	0.163882
	Error 1	12.4%	10.9%	−4.5%
	Error 2	10.8%	7.7%	−6.1%
	Error 3	−0.0009%	−26.3%	−5.6%
1	SSDPT [40]	1.062840	−2.406797	0.277018
	TSDPT [40]	1.077690	−2.473903	0.272347
	FSDPT [29]	1.190728	−3.471099	0.221768
	Present	1.194105	−2.678183	0.281900
	Error 1	12.4%	11.3%	1.8%
	Error 2	10.8%	8.3%	3.5%
	Error 3	0.3%	−22.8%	27.1%
3	SSDPT [40]	1.141655	−2.020416	0.269608
	TSDPT [40]	1.157693	−2.081815	0.270110
	FSDPT [29]	1.280741	−3.031284	0.257465
	Present	1.282283	−2.245913	0.260808
	Error 1	12.3%	11.2%	−3.3%
	Error 2	10.8%	7.9%	−3.4%
	Error 3	0.1%	−25.9%	1.3%
5	SSDPT [40]	1.154412	−1.957959	0.272070
	TSDPT [40]	1.170720	−2.018086	0.274512
	FSDPT [29]	1.296101	−2.956534	0.272062
	Present	1.296532	−2.176143	0.262983
	Error 1	12.3%	11.1%	−3.3%
	Error 2	10.7%	7.8%	−4.2%
	Error 3	0.04%	−26.4%	−3.3%

**Table 3:** Dimensionless center deflections $\bar{\tilde{w}}$ of the different symmetric FGM sandwich plates.

*s*	Theory	$\bar{\tilde{w}}$			
		1-3-1	1-4-1	1-5-1	1-6-1
0	Reissner	0.895427	0.895427	0.895427	0.895427
	Reddy	0.808168	0.808168	0.808168	0.808168
	Touratier	0.796783	0.796783	0.796783	0.796783
1	Reissner	1.058120	1.034178	1.016134	1.002128
	Reddy	0.954808	0.933207	0.916933	0.904303
	Touratier	0.941636	0.920308	0.904235	0.891760
3	Reissner	1.123332	1.089506	1.063861	1.043945
	Reddy	1.013647	0.983087	0.959932	0.941960
	Touratier	0.999831	0.969663	0.946795	0.929038
5	Reissner	1.142467	1.106027	1.078229	1.056585
	Reddy	1.322916	0.998014	0.972901	0.953358
	Touratier	1.016938	0.984428	0.959631	0.940325

**Table 4:** Dimensionless normal stress ${\bar{\tilde{\sigma }}}_{xx}$ of the different symmetric FGM sandwich plates.

*s*	Theory	${\bar{\tilde{\sigma }}}_{xx}$			
		1-3-1	1-4-1	1-5-1	1-6-1
0	Reissner	−2.650327	−2.650327	−2.650327	−2.650327
	Reddy	−2.461177	−2.461177	−2.461177	−2.461177
	Touratier	−2.388909	−2.388909	−2.388909	−2.388909
1	Reissner	−3.345092	−3.462328	−3.550618	−3.619105
	Reddy	−3.076466	−3.182234	−3.26186	−3.323619
	Touratier	−3.001256	−3.105682	−3.184311	−3.245294
3	Reissner	−3.026031	−3.191827	−3.317418	−3.414879
	Reddy	−2.788595	−2.938375	−3.051764	−3.139716
	Touratier	−2.716584	−2.864442	−2.976419	−3.063296
5	Reissner	−2.932290	−3.110958	−3.247136	−3.353091
	Reddy	−2.703191	−2.865312	−2.988331	−3.083995
	Touratier	−2.632905	−2.794581	−2.922533	−2.884668

**Table 5:** Dimensionless transverse shear stress ${\bar{\tilde{\tau }}}_{xz}$ of the different symmetric FGM sandwich plates.

*s*	Theory	${\bar{\tilde{\tau }}}_{xz}$			
		1-3-1	1-4-1	1-5-1	1-6-1
0	Reissner	0.163382	0.163882	0.163882	0.163882
	Reddy	0.174481	0.174481	0.174481	0.174481
	Touratier	0.171603	0.171603	0.171603	0.171603
1	Reissner	0.291248	0.287672	0.282126	0.275960
	Reddy	0.289538	0.286179	0.281109	0.275520
	Touratier	0.300347	0.296398	0.290494	0.284036
3	Reissner	0.312159	0.313772	0.310535	0.305124
	Reddy	0.308697	0.309871	0.306802	0.301833
	Touratier	0.322239	0.323475	0.319771	0.313921
5	Reissner	0.313283	0.317634	0.315957	0.311397
	Reddy	0.309879	0.313459	0.311757	0.307532
	Touratier	0.323573	0.327623	0.325487	0.320468

## Data Availability

Data sharing not applicable.

## Appendix A

The coefficient matrix $\left[\tilde{\Gamma }\right]$ is given by:

$$\begin{array}{l} {\tilde{\Gamma }}_{11}={\tilde{A}}_{11}{\tilde{\lambda }}^{2}+{\tilde{A}}_{66}{\tilde{\varsigma }}^{2},{\tilde{\Gamma }}_{12}=\tilde{\lambda }\tilde{\varsigma }\left({\tilde{A}}_{12}+{\tilde{A}}_{66}\right),{\tilde{\Gamma }}_{13}=-\tilde{\lambda }\left[{\tilde{A}}_{11}^{s}{\tilde{\lambda }}^{2}+\left({\tilde{A}}_{12}^{s}+2{\tilde{A}}_{66}^{s}\right){\tilde{\varsigma }}^{2}\right]\\ {\tilde{\Gamma }}_{14}=-\tilde{\lambda }\left[{\tilde{B}}_{11}^{1}{\tilde{\lambda }}^{2}+\left({\tilde{B}}_{12}^{s}+2{\tilde{B}}_{66}^{s}\right){\tilde{\varsigma }}^{2}\right],{\tilde{\Gamma }}_{22}=A_{66}{\tilde{\lambda }}^{2}+{\tilde{A}}_{66}{\tilde{\varsigma }}^{2},{\tilde{\Gamma }}_{23}=-\tilde{\varsigma }\left[{\tilde{A}}_{22}^{s}{\tilde{\varsigma }}^{2}+\left({\tilde{A}}_{12}^{s}+2{\tilde{A}}_{66}^{s}\right){\tilde{\lambda }}^{2}\right]\\ {\tilde{\Gamma }}_{24}=-\tilde{\varsigma }\left[{\tilde{B}}_{22}^{s}{\tilde{\varsigma }}^{2}+\left({\tilde{B}}_{12}^{s}+2{\tilde{B}}_{66}^{s}\right){\tilde{\lambda }}^{2}\right],{\tilde{\Gamma }}_{33}={\tilde{B}}_{11}{\tilde{\lambda }}^{4}+2\left({\tilde{B}}_{12}+2{\tilde{B}}_{66}\right){\tilde{\lambda }}^{2}{\tilde{\varsigma }}^{2}+{\tilde{B}}_{22}{\tilde{\varsigma }}^{4}\\ {\tilde{\Gamma }}_{34}={\tilde{C}}_{11}^{s}{\tilde{\lambda }}^{4}+2\left({\tilde{C}}_{12}^{s}+2{\tilde{C}}_{66}^{s}\right){\tilde{\lambda }}^{2}{\tilde{\varsigma }}^{2}+{\tilde{C}}_{22}^{s}{\tilde{\varsigma }}^{4},{\tilde{\Gamma }}_{44}={\tilde{C}}_{11}{\tilde{\lambda }}^{4}+2\left({\tilde{C}}_{12}+2{\tilde{C}}_{66}\right){\tilde{\lambda }}^{2}{\tilde{\varsigma }}^{2}+{\tilde{C}}_{22}{\tilde{\varsigma }}^{4}+{\tilde{E}}_{44}{\tilde{\varsigma }}^{4}+{\tilde{E}}_{55}{\tilde{\lambda }}^{4} \end{array}$$

The generalized force vector $\left[\tilde{F}\right]={\left[\begin{array}{cccc} {\tilde{F}}_{1} & {\tilde{F}}_{2} & {\tilde{F}}_{3} & {\tilde{F}}_{4} \end{array}\right]}^{T}$ can be written as:

$$\begin{array}{l} {\tilde{F}}_{1}=-\tilde{\lambda }\left({\tilde{A}}_{\tilde{T}}{\tilde{t}}_{1}+{\tilde{B}}_{\tilde{T}}{\tilde{t}}_{2}+B_{\tilde{T}}^{\alpha }t_{3}\right),{\tilde{F}}_{2}=-\tilde{\varsigma }\left({\tilde{A}}_{\tilde{T}}{\tilde{t}}_{1}+{\tilde{B}}_{\tilde{T}}{\tilde{t}}_{2}+B_{\tilde{T}}^{\alpha }{\tilde{t}}_{3}\right)\\ {\tilde{F}}_{3}={\tilde{q}}_{0}+H\left({\tilde{\lambda }}^{2}+{\tilde{\varsigma }}^{2}\right)\left({\tilde{B}}_{\tilde{T}}{\tilde{t}}_{1}+{\tilde{C}}_{\tilde{T}}{\tilde{t}}_{2}+{\tilde{C}}_{\tilde{T}}^{\alpha }t_{3}\right),{\tilde{F}}_{4}={\tilde{q}}_{0}+H\left({\tilde{\lambda }}^{2}+{\tilde{\varsigma }}^{2}\right)\left({\tilde{D}}_{\tilde{T}}{\tilde{t}}_{1}+{\tilde{E}}_{\tilde{T}}{\tilde{t}}_{2}+{\tilde{E}}_{\tilde{T}}^{\alpha }{\tilde{t}}_{3}\right) \end{array}$$

where

$$\begin{array}{c} \left(\begin{array}{c} {\tilde{A}}_{\tilde{T}}\\ {\tilde{B}}_{\tilde{T}}\\ {\tilde{C}}_{\tilde{T}} \end{array}\right)=\overset{3}{\underset{i=1}{\sum }}\int_{z_{i}}^{z_{i+1}}\frac{E^{\left(i\right)}}{1-{\left(\vartheta^{\left(i\right)}\right)}^{2}}\left(1+\vartheta^{\left(i\right)}\right)\alpha^{\left(i\right)}\left(\begin{array}{c} 1\\ \bar{z}\\ {\bar{z}}^{2} \end{array}\right)dz,\left(\begin{array}{c} {\tilde{B}}_{\tilde{T}}^{\alpha }\\ {\tilde{C}}_{\tilde{T}}^{\alpha } \end{array}\right)=\overset{3}{\underset{i=1}{\sum }}\int_{z_{i}}^{z_{i+1}}\frac{E^{\left(i\right)}}{1-{\left(\vartheta^{\left(i\right)}\right)}^{2}}\left(1+\vartheta^{\left(i\right)}\right)\alpha^{\left(i\right)}\bar{\tilde{\zeta }}\left(z\right)\left(\begin{array}{c} 1\\ \bar{z} \end{array}\right)dz\\ \left(\begin{array}{c} {\tilde{D}}_{\tilde{T}}\\ {\tilde{E}}_{\tilde{T}}\\ {\tilde{E}}_{\tilde{T}}^{\alpha } \end{array}\right)=\overset{3}{\underset{i=1}{\sum }}\int_{z_{i}}^{z_{i+1}}\frac{E^{\left(i\right)}}{1-{\left(\vartheta^{\left(i\right)}\right)}^{2}}\left(1+\vartheta^{\left(i\right)}\right)\alpha^{\left(i\right)}\bar{\tilde{k}}\left(z\right)\left(\begin{array}{c} 1\\ \bar{z}\\ \bar{\tilde{\zeta }}\left(z\right) \end{array}\right)dz \end{array}$$

in which

$$\bar{z}=\frac{z}{H},\bar{\tilde{\zeta }}\left(z\right)=\frac{\bar{\tilde{\zeta }}(z)}{H},\bar{\tilde{k}}\left(z\right)=\frac{\bar{\tilde{k}}(z)}{z}$$
